# Initial observations of medically complex young adults transitioning to adult care: Revealing data regarding mental health, nutrition, and transition preparedness

**DOI:** 10.1016/j.hctj.2023.100005

**Published:** 2023-06-21

**Authors:** Emily Hooker, Madeline Eckenrode, Betsy Hopson, Carlie Stein Somerville

**Affiliations:** a University of Alabama at Birmingham, Heersink School of Medicine, Birmingham, AL, USA; b Department of Medicine, University of Alabama at Birmingham, AL, USA; c Department of Neurosurgery, University of Alabama at Birmingham, AL, USA; d Division of Pediatric Neurosurgery, University of Alabama at Birmingham, AL, USA; e Department of Pediatrics, University of Alabama at Birmingham, AL, USA

**Keywords:** Transition care, Medical complexity, Interdisciplinary clinic, Mental health, Nutrition

## Abstract

**Background:**

Approximately one million adolescents with medical complexity transition into adult healthcare each year. Pediatric and adult providers alike have identified several barriers to transition, including insufficient knowledge of community resources, a lack of reimbursement for providing transition services, and an overall shortage of medical providers. Because of these barriers, the healthcare needs and challenges of this population have been understudied. The purpose of this article is to describe findings regarding nutrition, mental health, transition readiness, and caregiver burnout in a patient population of medically complex young adults in the interdisciplinary STEP clinic in the state of Alabama. Through the establishment of streamlined transition programs such as STEP, providers can address the unique health care needs of this patient population, with particular emphasis on optimizing mental health and nutrition.

**Methods:**

All patients seen at the STEP clinic from September 2020 to August 2021 (n = 169) were evaluated through pertinent validated assessments of transition readiness (TRAQ), and/or caregiver burden (ZBI), and/or mental health (PHQ9, GAD7). Additional variables collected from patient charts include age, race, diagnosis, primary insurance type, equipment dependence, and body mass index (BMI).

**Results:**

The average TRAQ score of patients included in this study is 3.57 ± 1.11. A TRAQ score of below 4.0 suggests that the patient is not yet ready to transition from pediatric to adult care. The average ZBI score of caregivers is 20.97 ± 12.59, which indicates mild to moderate caregiver burden. 33% of patients were found to have moderate to severe anxiety, and 28% were found to have moderate to severe depression. Only 3.6% of patients seen at the STEP clinic have a BMI within normal limits. 113 of our patients have ongoing use of medical technology, and 49% are wheelchair users.

**Conclusions:**

The need for organized transition care for medically complex young adults is apparent and growing. This patient population faces unique challenges in navigating the adult healthcare system, and patients are often unprepared for the transition. It is crucial that multidisciplinary clinics in which mental health and nutrition are addressed become accessible to all patients within this high-risk population.

## 1 Introduction

Advances in medical knowledge and the development of new technologies and therapeutic interventions have resulted in an increasing population of children and adolescents with chronic illnesses. A recent study reports that 26.6% of children ages 5–14 in the United States have some form of chronic illness or condition.^1^ It is estimated that one million children with special healthcare needs transition to adult care each year.^2^ Providers face many challenges in guiding youth with complex medical problems as they transition from pediatric to adult health care. According to recent studies, pediatricians report several barriers to transition, including a shortage of adult providers, lack of reimbursement for providing transition services and counseling, and inadequate education about community support services.^3^ Adult providers describe a lack of training on caring for patients with chronic diseases of childhood, insufficient communication between the pediatric and adult providers, and difficulties caring for patients with complex medical needs without involvement of the patients’ families, especially when discussions of guardianship have not occurred.^3^ Despite the apparent deficits in transition care, there is a paucity of established clinical approaches and tools for success available to both pediatricians and adult providers.

In 2012, the state of Alabama was home to 69,400 children ages 0–20 years old who were reported to have one or more disabilities, including visual, hearing, ambulatory, cognitive, self-care, and independent living disabilities.^4^ By 2020, Alabama was noted to have the fourth highest percentage of individuals living with disabilities, with 16.7% of the population having at least one disability compared with the national average of 12.6%.^5^ Additionally, Alabama faces unique healthcare challenges given the high rates of obesity, chronic disease, lack of insurance, and poverty. These factors contribute to a high-risk environment for poor health outcomes and emphasize the need for accessible, multidisciplinary transition care for children with chronic illnesses.^6^

It has been well-established that children and adolescents with medical complexity are at increased risk of mental health disorders, which negatively impact patient compliance and health outcomes.^7^ Children and adolescents with chronic illnesses are more likely to experience peer rejection and inappropriate parenting behaviors, which negatively impact self-esteem and contribute to both anxious and depressive symptoms.8., 9. Additionally, patients may experience distressing side-effects associated with medications necessary to manage their chronic conditions, which also negatively impact self-concept and mental health.^8^ Those who experience life-threatening symptoms of illness, such as shortness of breath or seizures, are significantly more likely to report symptoms of anxiety.^9^ The high prevalence of mental health problems in adolescents with chronic illnesses and disabilities must be considered when providing holistic primary care to these patients.

The STEP (“Staging Transition for Every Patient”) program was developed as a joint effort between Children’s of Alabama (COA) and the University of Alabama at Birmingham Health System (UAB) to meet the growing need for transition care for medically complex young adults. Medical complexity is defined by the American Academy of Pediatrics (AAP) as a combination of multiorgan involvement due to chronic medical condition(s), functional impairment, ongoing dependence on medical technology, and high resource need.^10^ The STEP clinic at UAB accepts referrals for patients ages 18 and above, with no upper age limit. Referrals are most often from pediatric subspecialists but can also be from pediatricians or other primary care providers, self-referrals from patients or families, or adult healthcare providers. At the time these data were collected, there was only one inclusion criterion, which was that the patient needed to have a childhood-onset chronic medical condition that persisted into adulthood. Since that time, inclusion criteria have been expanded and now include patients with at least two out of the following three criteria: intellectual disability and or autism; involvement of at least 3 organ systems in their disease process; and technology-dependence. The American Psychiatric Association defines intellectual disability as impairment in adaptive functioning within social, conceptual, and practical domains.^11^ The STEP program is the first of its kind in Alabama and one of only a handful of similar programs nationwide.

This paper details the demographics of the first cohort of patients seen at the STEP clinic as well as novel findings regarding the mental health and nutritional challenges faced by these patients. These findings highlight key areas for advancement relevant to all adult primary care providers who will care for this growing patient population.

## 2 Methods

### 2.1 Clinic development

To better understand transition-related needs within our hospital system, we conducted a needs assessment involving 18 specialty divisions at Children’s of Alabama. The results of this needs assessment are discussed further in a previously published paper describing the development of the STEP clinic.^12^

The STEP clinic opened its doors in September of 2020 with the goal of serving as a temporary clinical home for patients 18 years and older with chronic diseases of childhood. The clinic is staffed by physicians trained and licensed in Internal Medicine and Pediatrics (Med-Peds), as well as a social worker and physical therapist. Our nursing coordinator is also a crucial member of our team who oversees appointment scheduling and patient triage and assists with in-clinic procedures, such as urine sample collection and gynecologic examinations. We used information gathered in our needs assessment to identify patient populations who face the greatest challenges in transitioning. To improve transition care, we partnered with specialists from across UAB to hold diagnosis-specific interdisciplinary clinics. In total, we have five specialists embedded within our clinic, including pulmonary, transplant nephrology, physical medicine and rehabilitation, and both neuromuscular neurology and epilepsy neurology.

### 2.2 Demographic data

Our clinic nursing and physician staff collect information about every patient’s gender, age, insurance coverage, primary diagnoses, communication status, equipment needs, and current number of specialty providers, which are recorded in the electronic health record. While in the clinic, nursing staff measure and record patients’ BMI and the number of referrals made after a clinic visit. In addition, our clinic program director calculates the number of patients seen and the no-show rate for each clinic day, and these numbers are documented in a shared internal database.

### 2.3 Clinic assessments and screenings

Each patient seen in the STEP clinic receives a series of individualized assessments based on their medical conditions and level of independence. We ask our patients who are developing autonomy to complete the Transition Readiness Assessment Questionnaire (TRAQ), a validated, patient-centered questionnaire assessing patients’ ability to navigate the healthcare system independently.13., 14. Many of our patients have cognitive and/or physical challenges that affect their ability to complete surveys. In these cases, we ask that the patient’s partner or caregiver attending the visit assist the patient in completing the TRAQ, if applicable. The TRAQ gauges transition readiness by asking patients about their level of comfort with performing certain tasks, such as making their own appointments and refilling their medications, as well as more complex questions regarding the patients’ understanding of their insurance coverage and confidence in discussing their health with providers.^13^ It is important to note that independent function is not a goal of some of our patients due to severe physical or intellectual disability. If a patient is totally caregiver-dependent, we ask the patient’s caregiver to instead complete the Zarit Burden Interview (ZBI), a validated, 22-item assessment designed to describe physical, psychological, and economic stressors associated with full-time caregiving.15., 16. Due to the high prevalence of mental health problems among patients with chronic illness and disability, we also conduct both depression and anxiety screening using the Patient Health Questionnaire-9 (PHQ-9) and the General Anxiety Disorder-7 (GAD-7). These assessments, as well as discussion in the initial clinic visit, help us construct an individualized transition plan (ITP) for each patient, which develops specific goals for patients and caregivers to work on between visits.^12^ In patients who hope to develop autonomy, a low TRAQ score prompts goal setting as part of their ITP.

### 2.4 Analysis plan

IRB approval was obtained to do a retrospective chart review after the first year of clinical operations. Consent was waived due to the retrospective nature of the review and data was de-identified prior to analysis. Descriptive statistics were performed using IBM SPSS Statistics for MAC, version 27 (IBM Corp, Armonk, NY, USA). Counts and frequencies of screening assessments, body mass index, number of referrals, missed visits, communication status, and specialists are reported. Since this is a descriptive/observational study, there is no control group and therefore no p-values provided for comparisons of significance.

## 3 Results

### 3.1 Demographic data

The STEP clinic opened in September of 2020, and in its first year of operation, there were 169 new patients seen over a total of 267 visits (Table 1). The individuals seen had an age range of 18–42 years, with a mean age of 22.2 ± 4.1 years. There were 13 patients seen who were over the age of 30 years old. The racial distribution was similar to the current Alabama distribution, with 61% of patients identifying as white, 36% as Black, and 3% as other.^17^ 51% of patients were male. Over 99% of the patients seen were insured, with 47% reporting Medicaid as their primary insurance, 38% reporting BlueCross BlueShield, a private insurance product, and 8% reporting Medicare. A wide range of diagnoses were seen, but the four diagnoses with the greatest representation among STEP patients include cerebral palsy (25%), genetic disease (14%), neural tube defect (12%), and neurologic disease (11%).

**Table 1:** Patients seen in the first year of opening the STEP clinic (n = 169).

Parameter		n	%	M	SD
Gender					
	Female	82	49		
	Male	87	51		
Race					
	White	104	61		
	Black	60	36		
	Other	5	3		
Age				22.20	4.11
Primary Insurance					
	BCBS	65	38		
	Medicaid	74	44		
	Medicare	13	8		
	Self-pay	2	1		
	Other	15	9		
Secondary Insurance					
	BCBS	5	3		
	Medicaid	53	31		
	Other	2	1		
	None	109	64		
Primary Diagnosis					
	Autism spectrum	12	7		
	Autoimmune disorder	6	4		
	Bone marrow transplant	1	0.6		
	Congenital anomaly	6	4		
	Cerebral palsy	43	25		
	Diabetes mellitus	8	5		
	Genetic disorder	24	14		
	Neurologic disorder	18	11		
	Neuromuscular disorder	9	5		
	Renal disease	18	11		
	Spina bifida	21	12		
	Spinal cord injury	2	1		
	Severe ADHD	1	0.6		
Communication	Verbal	117	69		
	Non-verbal	52	31		
Equipment needs		113	67		

Fifty-two patients are non-verbal, while 117 patients are verbal. At least 73 patients have an intellectual disability, ranging from mild to severe, which represents 43% of our patient panel. It is important to note that a formal diagnosis of intellectual disability is not always included in patients’ medical records, and the accurate number of our patients with intellectual disability is likely greater than 73.

One hundred and thirteen of our patients are dependent on medical technology. For example, 82 patients are wheelchair users, 32 patients have a ventriculoperitoneal (VP) shunt, and 23 patients receive nutrition through a gastrostomy tube. These numbers represent 49, 19, and 14% of our patients, respectively.

The no-show rate for patient appointments was less than 10%. The percentage of telehealth visits was approximately 10%.

### 3.2 Assessments and screenings

Thirty-nine percent of our patients who are developing autonomy completed a TRAQ, with an average score of 3.57 ± 1.11. Of our patients who are considered totally caregiver dependent, 22% completed a ZBI, with an average score of 20.97 ± 12.59. A TRAQ score of less than 4.0 suggests that a patient is not yet ready to transition from pediatric to adult healthcare.^13^ A ZBI score of 21–40 is indicative of mild to moderate caregiver burden, while a score of 0–20 suggests little to no caregiver burden.^15^

Forty-two percent of patients completed the GAD-7, and of those, 33% received a score indicative of moderate to severe anxiety. 44% of patients completed the PHQ-9, and of those, 28% received a score indicative of moderate to severe depression. Patients seen via telemedicine or those who are nonverbal did not complete a GAD-7 or PHQ-9 due to staff time limitations.

The results of all assessments and screenings are summarized in Table 2.

**Table 2:** Results of assessments and screenings.

Parameter		n	%	M	SD
GAD-7		71	42	6.90	7.06
	Minimal (0–4)	39	54		
	Mild (5–9)	9	13		
	Moderate (10–14)	9	12		
	Severe (15–21)	15	21		
PHQ-9		74	44	5.89	6.45
	None to minimal (0–4)	40	54		
	Mild (5–9)	13	18		
	Moderate (10–14)	14	19		
	Moderately severe (15–19)	3	4		
	Severe (20–27)	4	5		
ZBI		37		20.97	12.59
	Little to no burden (0–21)	21	57		
	Mild to moderate burden (21–40)	14	38		
	Moderate to severe burden	(41–60)	2	5.4	
	Severe burden (61–88)	0	0		
TRAQ		66		3.57	1.11
BMI		141	83	25.61	8.51
	Underweight (< 18)	25	18		
	Normal weight (18–25)	5	3		
	Overweight (> 25)	63	45		
	Obese (> 30)	35	25		
	Morbidly obese (> 40)	13	9		

### 3.3 Nutritional status

Additionally, only 3.6% of patients seen at the STEP clinic have a BMI within normal limits. 17.7% of patients have a BMI that is considered underweight, 44.7% are considered overweight, 24.8% are considered obese, and 9.2% have a BMI within the range of class III obesity (a BMI greater than 40 kg/m^2^).^18^

## 4 Discussion

The data collected through the STEP program is the first data set representative of young adults with medical complexity in the state of Alabama, and the findings are truly sobering. The young adults in our clinic are facing serious mental health issues, malnutrition resulting in both low weight and obesity, and the reality that the adult healthcare system is not yet prepared to meet their needs. The experience and information gleaned from our first year is shaping the agenda for future programming and resource allocation, and we hope that it will contribute to further research on effective transition planning for medically complex youth.

The mental health concerns of our patient population are considerable. These patients are more likely than young adults without chronic diseases to suffer from anxiety and depression.^19^ Although Medicare requires annual depression screening for patients covered by Medicare, this only represents a small number of people younger than 65 (and, in our patient population, only 7% of patients). Traditional adult health practices may or may not screen annually for anxiety and depression. However, we found that mental health screening has identified a large percentage of patients with previously undiagnosed and/or untreated anxiety disorders and depression. Because this was an observational study, we do not have control groups to which to compare TRAQ, ZBI, PHQ-9, and GAD-7 scores. We do know that, nationally, based on 2022 survey data collected by the Kaiser Family Foundation, about one third of adults under thirty years old rated their mental health as “fair” or “poor”.^20^ Although our data and national results are certainly not perfect comparators, we can see that in many young adults in our country, mental health concerns are common.

When we identify patients with untreated or undertreated mental health concerns, we utilize shared decision-making to develop a treatment plan, which usually consists of either starting or modifying pharmacotherapy and making referrals for psychotherapy. Unfortunately, there is limited access to mental health resources throughout the state. Our findings emphasize the need for creating innovative strategies and partnerships with counseling and psychiatry resources for young adults with medical complexity. With improvements in the mental health of our patient population, we anticipate improvements in their overall health and ability to function independently.

In addition to higher rates of mental health disorders, young adults with chronic illness in our clinic have higher rates of obesity and protein calorie malnutrition than young adults in the general population.21., 22. We hypothesize that decreased mobility, dietary limitations, and social determinants of health are contributing to the growing obesity rates. The factors contributing to protein calorie malnutrition are more complex and include a higher incidence of oropharyngeal dysphagia and oral motor dysfunction in these patients, as well as a lack of adequate training for adult providers regarding nutrition and proper enteral nutrition management.23., 24. Referral options for adult GI providers with knowledge and training in this patient population are limited. However, we have formed a partnership with a palliative nutritionist who works with our patients with low BMI status, and we refer patients with obesity to our university’s adult weight management program. Nutrition optimization is very important for long-term health, and we plan to incorporate a registered dietician into our clinic during our next phase of expansion and hiring. We also plan to expand our telemedicine services to incorporate access to mental health and nutrition resources while minimizing cost and barriers to care for our patients and their families.^25^

The TRAQ scores of our patients indicate that many are not prepared to transition to the adult healthcare system. This assessment provides insight into which areas of transition would most benefit from targeted intervention. For example, if the patient has a low score in the category of managing medications, our team will work closely with the patient and caregiver to identify barriers the patient is facing and brainstorm solutions to overcome those barriers. Our intention is that patients will complete the TRAQ annually so that we can record patients’ progress in transition readiness. Similarly, we use the results of the ZBI to identify areas in which caregivers would benefit most from additional support and resources. Although most caregivers for our patient population report little to no caregiver burden, the ZBI allows us to identify the caregivers who report higher levels of burden and connect them with appropriate community resources. We also plan to administer the ZBI annually to track scores over time.

Our patients also represent a wide age range, which supports our belief that these patients have inadequate access to transition care. We have found that patients and their families are struggling with the transition process decades after the patients age out of pediatric care. This is why we have not included age in our exclusion criteria, and we anticipate that we will continue to see patients in their 30s and 40s until great improvements have been made in healthcare transitions.

It is important to note the limitations of using written screening tools in our patient population, given an estimated 75% prevalence of at least mild-to-moderate intellectual disability. However, we discuss with the patients and caregivers that screening tools are meant to reflect the patient experience, even if the patient requires assistance in completing them. We also have lower than anticipated completion rates of these screening tools, which we believe was influenced by several factors. Some patients were simply unable to complete them, while others were never given the screening tools to complete. Additionally, we are a single center reporting data which may not be translatable to other centers. Our results may also be impacted by the existence of a transition program for spina bifida within our center, whereas other centers without transition programs are likely caring for this patient population within the pediatric health care setting.^26^ Finally, we are located in a resource-limited state, which likely contributes to the prevalence of mental health and nutritional needs demonstrated among our patients. These limitations highlight the need for further exploration of best practices for caring for patients with chronic diseases of childhood as they transition from pediatric to adult health care within a broad spectrum of settings.

Comparing TRAQ, GAD-7, and PHQ-9 scores between patient groups within STEP is a future area of research. As our clinic population grows, we hope to be able to provide meaningful between group comparisons based on age, race, diagnosis, and other factors to target our efforts most effectively.

## 5 Conclusions

The need for organized transition care for medically complex young adults is apparent within our state. We are working to address that need through the development of the STEP program and clinic. STEP now serves as a bridge between Children’s of Alabama and UAB Hospital for all patients with complex conditions of childhood as they transition from pediatric to adult health care. Through the implementation of the STEP program, it has become clear that our patients face unique barriers to optimizing their health. We have seen that young adults with chronic illness are at higher risk for anxiety and depression and should be screened annually. Additionally, young adults with chronic illness experience difficulties maintaining a healthy body mass. Overall, we have observed the great need for better access to quality mental health and nutrition resources. To provide our patients with these life-saving resources, we must continue building partnerships between pediatric and adult providers, ensuring that providers receive necessary training and education, and calling attention to the need for a dramatic change in managing the health of this vulnerable population.

## Conflicts of Interest

The authors have no conflicts of interest to disclose.

## Funding

**The author(s) disclosed receipt of the following financial support for the research, authorship, and/or publication of this article:** This work was supported by the NIH NHLBI T35 Summer-Term Health-Related Training in Professional Schools (Grant no. 5T35HL007473).

## Ethical Statement

Hereby, I, Emily Hooker, consciously assure that for the manuscript /insert title/ the following is fulfilled:

2) This material is the authors' own original work, which has not been previously published elsewhere.
3) The paper is not currently being considered for publication elsewhere.
4) The paper reflects the authors' own research and analysis in a truthful and complete manner.
5) The paper properly credits the meaningful contributions of co-authors and co-researchers.
6) The results are appropriately placed in the context of prior and existing research.
7) All sources used are properly disclosed (correct citation). Literally copying of text must be indicated as such by using quotation marks and giving proper reference.
8) All authors have been personally and actively involved in substantial work leading to the paper, and will take public responsibility for its content.

The violation of the Ethical Statement rules may result in severe consequences.

To verify originality, your article may be checked by the originality detection software iThenticate. See also http://www.elsevier.com/editors/plagdetect.

I agree with the above statements and declare that this submission follows the policies of Health Care Transitions as outlined in the Guide for Authors and in the Ethical Statement.

## Declaration of Competing Interest

The authors declare that they have no known competing financial interests or personal relationships that could have appeared to influence the work reported in this paper.

## Data Availability

The data that has been used is confidential.

## References

[1] Kish A.M., Newcombe P.A., Haslam D.M. (2018). Working and caring for a child with chronic illness: a review of current literature. Child: Care Health Dev.

[2] Cheng L, Shaewitz D., The 2021 Youth Transition Report: Outcomes for Youth and Young Adults with Disabilities. Washington, DC: Institute for Educational Leadership.

[3] White P.H. (2012). A primary care quality improvement approach to health care transition. Pediatr Ann.

[4] Erickson W., Lee C., von Schrader S. (2012).

[5] U.S. Disability Statistics by State, County, City, and Age; 2017 [cited 2021 July 9]; Available from: 〈https://www.disabled-world.com/disability/statistics/scc.php#state〉.

[6] Grumbach K. (2003). Chronic illness, comorbidities, and the need for medical generalism. Ann Fam Med.

[7] Delamater A.M., Guzman A., Aparicio K. (2017). Mental health issues in children and adolescents with chronic illness. Int J Hum Rights Healthc.

[8] Pinquart M., Shen Y. (2011). Depressive symptoms in children and adolescents with chronic physical illness: an updated meta-analysis. J Pediatr Psychol.

[9] Pinquart M., Shen Y. (2011). Anxiety in children and adolescents with chronic physical illnesses: a meta-analysis. Acta Paediatr.

[10] Kuo D.Z., Houtrow A.J., Council On Children With D. (2016). Recognition and management of medical complexity. Pediatrics.

[11] Anon (2013). Diagnostic and Statistical Manual of Mental Disorders.

[12] Hopson B. (2022). The development of a transition medical home utilizing the individualized transition plan (ITP) model for patients with complex diseases of childhood. Disabil Health J.

[13] Transition Readiness Assessment Questionnaire. [cited 2021 July 10]; Available from: 〈https://www.etsu.edu/com/pediatrics/traq/〉.

[14] Wood D.L. (2014). The Transition Readiness Assessment Questionnaire (TRAQ): its factor structure, reliability, and validity. Acad Pediatr.

[15] Adib-Hajbaghery M., Ahmadi B. (2019). Caregiver burden and its associated factors in caregivers of children and adolescents with chronic conditions. Int J Community Based Nurs Midwifery.

[16] Bachner Y.G., O'Rourke N. (2007). Reliability generalization of responses by care providers to the Zarit Burden Interview. Aging Ment Health.

[17] QuickFacts Alabama. United States Census Bureau; 2022.

[18] Weir CB, JA. BMI Classification Percentile and Cut off Points. StatPearls; 2022 [cited 2023 May 10]; Available from: 〈https://www.ncbi.nlm.nih.gov/books/NBK541070/〉. 31082114

[19] Thomas J. (2003). A descriptive and comparative study of the prevalence of depressive and anxiety disorders in low-income adults with type 2 diabetes and other chronic illnesses. Diabetes Care.

[20] Lopes L, et al. KFF/CNN Mental Health in America Survey; 2022 [cited 2023 May 13]; Available from: 〈https://www.kff.org/report-section/kff-cnn-mental-health-in-america-survey-findings/〉.

[21] Overweight & Obesity Statistics; 2021 [cited 2023 14 June]; Available from: 〈https://www.niddk.nih.gov/health-information/health-statistics/overweight-obesity〉.

[22] Adults Aged ≥ 15 Years Who Are Underweight (%). Indicator Metadata Registry List [cited 2023 14 June]; Available from: 〈https://www.who.int/data/gho/indicator-metadata-registry/imr-details/370#:∼:text=About%203%2D5%25%20of%20a,m%C2%B2)%20among%20total%20adult%20population〉.

[23] Trivic I., Hojsak I. (2019). Evaluation and treatment of malnutrition and associated gastrointestinal complications in children with cerebral palsy. Pediatr Gastroenterol Hepatol Nutr.

[24] Kumpf V.J., Tillman E.M. (2012). Home parenteral nutrition: safe transition from hospital to home. Nutr Clin Pract.

[25] Shore J.H. (2018). Best practices in videoconferencing-based telemental health april 2018. Telemed J E Health.

[26] Hopson B. (2018). The development of a lifetime care model in comprehensive spina bifida care. J Pediatr Rehabil Med.

